# A Qualitative Study of Characteristics of an Effective Health Coach: Personal, Professional, and Program Based

**DOI:** 10.1093/geroni/igab046.1454

**Published:** 2021-12-17

**Authors:** Caitlin Clason, Frances Barg, Barbara Riegel

**Affiliations:** 1 University of Pennsylvania, Philadelphia, Pennsylvania, United States; 2 University of Pennsylvania Perelman School of Medicine, Philadelphia, Pennsylvania, United States

## Abstract

Health coaching continues to grow in popularity as an effective intervention to empower and engage patients and their caregivers. However, little is known about what characteristics contribute to the success of health coaches in implementing evidence-based interventions. This study examines the characteristics that contribute to effective health coaches. Semi-structured interviews were conducted with health coaches and an interdisciplinary research team of an ongoing study examining a virtual health coaching intervention. Interviewees identified three discrete themes of characteristics that contribute to the success of health coaches: personal (e.g. compassion), professional (e.g. transferability of soft skills) and program based (e.g. training regimen). We conclude that it is not just innate personality attributes that make a health coach effective in their role, but training and program design intended to support health coaches are also important in implementing interventions.

